# A Crest Factor Reduction Technique for LTE Signals with Target Relaxation in Power Amplifier Linearization

**DOI:** 10.3390/s22031176

**Published:** 2022-02-04

**Authors:** José Ricardo Cárdenas-Valdez, Jose Alejandro Galaviz-Aguilar, Cesar Vargas-Rosales, Everardo Inzunza-González, Leonardo Flores-Hernández

**Affiliations:** 1 IT de Tijuana, Tecnológico Nacional de México, Tijuana 22435, Mexico; jose.cardenas@tectijuana.edu.mx (J.R.C.-V.); leonardo.flores201@tectijuana.edu.mx (L.F.-H.); 2 School of Engineering and Sciences, Tecnologico de Monterrey, Monterrey 64849, Mexico; cvargas@tec.mx; 3 Facultad de Ingeniería, Arquitectura y Diseño, Universidad Autónoma de Baja California (UABC), Carret. Transpeninsular Ensenada-Tijuana No. 3917, Fracc. Playitas, Ensenada 22860, Mexico; einzunza@uabc.edu.mx

**Keywords:** crest factor reduction, FPGA, LTE, PAPR, power amplifier

## Abstract

The signal conditioning treatment to achieve good relation of power with radio-frequency (RF) conversion in conventional transceiver systems require precise baseband models. A developed framework is built to provide a demonstration of the modeling figures of merit with orthogonal frequency division multiplexing (OFDM) support under signal conditioning and transmission restrictions to waveforms with high peak to average power ratio (PAPR) in practical applications. Therefore, peak and average power levels have to be limited to correct high PAPR for a better suited correction power from the amplifier that can lead to compression or clipping in the signal of interest. This work presents an alternative joint crest factor reduction (CFR) algorithm to correct the performance of PAPR. A real-time field-programmable gate array (FPGA) testbed is developed to characterize and measure the behavior of an amplifier using a single-carrier 64–QAM OFDM based on long-term evolution (LTE) downlink at 2.40 GHz as stimulus, across wide modulation bandwidths. The results demonstrate that the CFR accuracy capabilities for the signal conditioning show a reliable clipping reduction to give a smooth version of the clipping signal and provide a factor of correction for the unwanted out-of-band emission validated according to the adjacent channel power ratio (ACPR), PAPR, peak power, complementary cumulative distribution function (CCDF), and error vector magnitude (EVM) figures of merit.

## 1. Introduction

In modern wireless telecommunications systems, the power amplifier (PA) is a key device in terms of energy consumption. Thus, becomes crucial to provide new techniques to overcome the tradeoff between efficiency and linearity. The PAs are the most critical components in a radio-frequency (RF) transmission system, mostly because they add most short-term and long-term memory effects during a digital information transmission, producing intermodulation products and spectral regrowth in adjacent channels. Memory effects are of two types of short and long term, caused by the biasing circuit or inherent to the active device itself as thermal effects; any low-frequency dispersion effects will manifest as envelope memory affecting the PA performance. Both types can be modeled as a black box based on behavior modeling with memory [1]. To improve the performance in linearity and power efficiency in data waveforms, crest factor reduction (CFR) in conjunction with digital predistortion (DPD) as linearization technique are used [2]. In [3], a novel multi-objective predistortion linearization approach based on a learning procedure constrained algorithm with prescribed linearity levels is able to maximize the RF output power. In the DPD modeling, a CFR technique is usually implemented to apply a correction to reduce significant peaks in the baseband signal.

To handle the baseband waveforms, digital multiplexing formats such as orthogonal frequency division multiplexing (OFDM) require an extensive range of data to signals used in wide code division multiple access (W-CDMA), and long-term evolution (LTE), which is the standard used for broadband 5G New Radio (NR) communication networks [4], or as the proposed predistortion algorithm for peak to average power ratio (PAPR) reduction in OFDM transmission, as in [5] without a feedback loop. Due to the demand for high bandwidth efficiency from 20 to 30-MHz [6], and the need for power efficiency, the usage of the DPD techniques offer digital flexibility to linearize the undesired behavior of the PAs. Applications in the 2 GHz band is a current topic, whose techniques are migrating towards 5G NR, applications for 10-MHz LTE bandwidths, so it is essential to develop methodologies that evaluate the error vector magnitude (EVM) and adjacent channel power ratio (ACPR) achieved [7].

An additional work is considered for the evaluation of a system with a memoryless model to reduce the PAPR, thus improving the spectral efficiency based on the signaling technique faster than Nyquist clipping multicarrier [8]. In the same way, a new model for the theoretical calculation of harmonics based on clipping has been developed in order to design loads for positive high-frequency devices to avoid non-linearity effects of second or third order [9]. In [10], an interference-aware iterative clipping and filtering PAPR reduction scheme is developed for multiple-input–multiple-output (MIMO)-OFDM systems. Additionally, in [2], a complete study of existing linearization techniques that shows the aspects to consider into a 5G NR, particularly with W-CDMA and 64–QAM stimulus signals through several methods, such as the feedforward, feedback, and predistortion, is introduced.

Additional efforts related to linearization and spectral regulation have been made for LTE and W-CDMA, a cubic spline (CS) has been developed for a DPD system for the outphasing angles and power level correction applied to Class-F PA for a W-CDMA 5-MHz and an LTE 10-MHz [11]. Additionally, a numerically stable digital predistorter for RF PA linearization based on Gegenbauer polynomials has been implemented for PA output under 4-channel W-CDMA and LTE drive signals [12]. An interesting feature to provide real-time prototyping systems for developing new DPD approaches is offered with software-defined radio (SDR), which is a favorable solution to address such problems. SDR is a rapidly developing technology in the telecommunication industry to provide flexible and reconfigurable transceiver architectures. Thus, the implementation of FPGA platforms in addition to ARM processors can be enabled to reconfigure a portion of hardware algorithms to process different communication standards and conditioning signal algorithms in a flexible test platform [13].

In systems that operate in the 2-GHz band and 5G NR, the PAPR brings another restriction related to spectral efficiency due to the back off of the PA and its operating point, which must be greater than the signal introduced to the PA to avoid saturation [14,15], CFR is used to improve the spectral efficiency for concurrent bands in terms of efficiency and power handling [7], the main function of a CFR analysis stage in the transmitter stage is to improve the spectral efficiency for concurrent bands in term of power management [16]. The clipping technique is developed based on limiting the signal to the desired power level in the transmitter without changing the phase. In this work, an implementation with an FPGA evaluation board was developed. It was demonstrated that a CFR technique aimed at addressing PAPR reduction for LTE commercial RF PAs. It is important to note that in LTE systems, a large PAPR is not such a problem for signal transmission, however, it has a direct impact on the efficiency of the RF PA at the transmitter.

In order to compensate the receiver side of the transmitter impairment, some works involve the clipping technique. Various works on PAPR reduction techniques have been presented in the literature, in [17], for OFDM-based digital multiplexing, validating the results with a PA for ACPR less than −55 dBc. Ref. [18] explores clipping and filtering applied to single-carrier and two-carrier 20-MHz LTE signals as the stimulus, the developed CFR technique improves the spectral regrowth using a testbench of a device under test (DUT) operating at 3.3–3.8 GHz. This work focuses on the design and implementation stage of a transceiver that operates in the 2 GHz band for LTE applications. In the implementation stage, clipping-based signal analysis is used to reduce the PAPR of the system, in addition to CFR for managing the PAPR which is described by the complementary cumulative distribution function (CCDF) statistical measure of the peak power. In [19,20], the clipping method is used to compensate the output of the transmitter system using a correlation factor to minimize the floor error, based on the transformation of the amplitude to a defined distribution, using a conventional clipping process, followed by filtering. In [21] an analytical work based on the reconstruction of iterative clipping is presented; this scheme performs the reconstruction of the non-linear distortion of a digital multiplexing system such as OFDM. In [22], experimental results applied to a WiMax 802.16e transmitter are presented, which through the clipping process improves the energy use of the amplification stage. Additionally, in [23], a clipping application for Internet of things (IoT)-based 5G or IoT-based machine type communication is presented to link a low-cost transmitter with a high-level receiver and compensate for the imbalances.

The remainder of this paper is organized as follows: Section 2 introduces the CFR technique related to the joint PAPR clipping. Experimental results to demonstrate the performance of the aforementioned CFR algorithm are discussed in Section 3. In addition, further details about the testbed setup used for the specific experimental tests validation of the CFR method and predistortion model are given in Section 4. Finally, in Section 5 the conclusion and future work are given.

## 2. Signal Modeling and CFR Method

**Figure 1:**
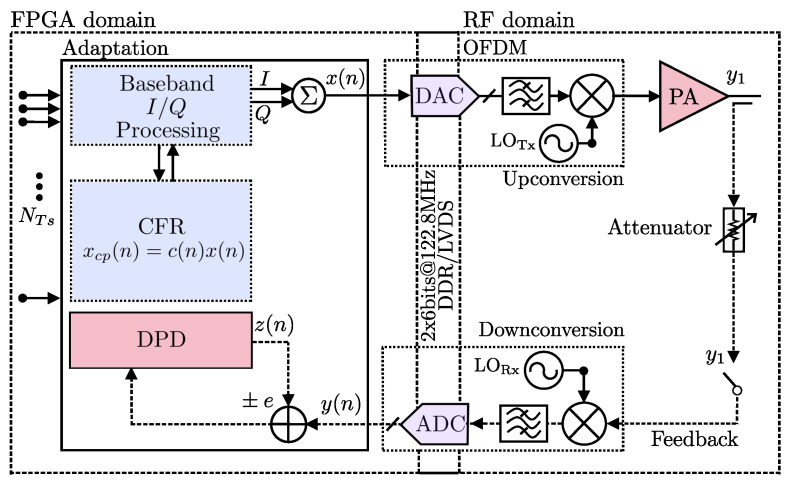
Block diagram for the SDR downlink system process with the feedback observation path up-conversion/down-conversion.

A block diagram of an SDR downlink is depicted in Figure 1, which presents a system able to support the real-time baseband signal generation with several bandwidths. The front-end system-level analysis for specific application and implementation with a digital signal processing approach, provides the flexible signal treatment between domains (Digital-FPGA to RF) as shown in Figure 1. Furthermore, more issues related to complex gain variations (amplitude and phase), in the RF front end (RFFE) performance are considered in three effects: the amplitude and frequency distortion, and memory effects to modeling the signal properties. So that, in this investigation the SDR transceiver implementation consider three key aspects: (*i*) modulated signal generation, (*ii*) feedback response of the DUT, and algorithm correction for (*iii*) CFR and offline predistortion (PD) calculation. Thus, the input x(n) and output y(n) of the DUT are used to calculate the signal properties such as amplitude and phase distortions added in the RF domain. Thus, the signals are calculated to evaluate the figures of merit of the system and are generated by the DUT in order to adapt the algorithms in terms of CFR, PAPR, gain, and further to obtain the predistortion function.

### 2.1. CFR Model Identification with Joint PAPR Clipping

**Figure 2:**
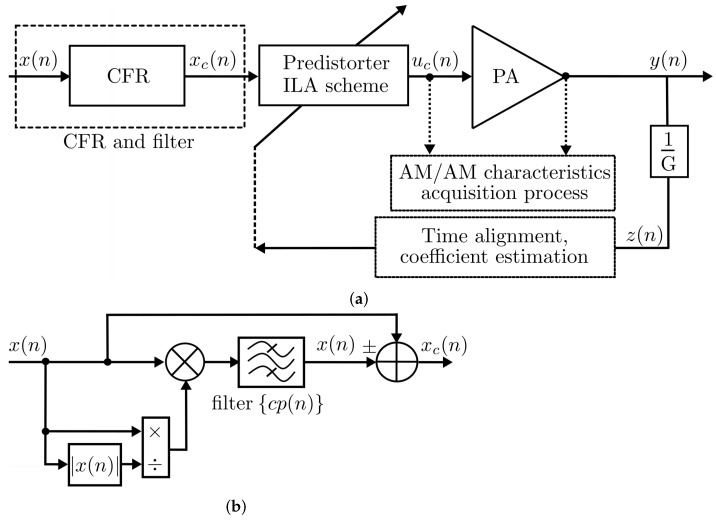
Block diagram of the closed-loop behavioral modeling process with CFR: (**a**) ILA for linearization with CFR clipping reduction, (**b**) CFR and filter process.

The CFR model identification algorithm is examined to establish a PAPR reduction for LTE 5-MHz and LTE 10-MHz bandwidth applications, and it is summarized in this section. The development of a platform for LTE applications based on a combination of linearization with CFR from estimation of instantaneous PAPR is able to estimate the maximum power of a sample in an OFDM transmitter symbol divided by the average OFDM power. This condition considerably improves the phase shifts that may exist between subcarriers of a transceiver system. In Figure 2a, it is shown a generalized structure cascade connected using a conventional computation of CFR and the PD model extraction. For this system, the transceiver signal is closed-loop feedback with y(n) being the PA output, x(n) as input signal in addition to the CFR stage known in clipping stages that together with the indirect learning approach (ILA) scheme generates a new u(n) to characterize the amplitude to amplitude (AM–AM) conversion curve of the DUT. Thus, the final signal $y_{d}\left(n\right)$ can be generated from the inverse gain estimate, and correction for this process is crucial for the $x_{c}\left(n\right)$ as shown in Figure 2b.

As mentioned before, for LTE applications with a variation of the clipping level, the benefit to remove preliminary unwanted distortion of the system, can help initially to operate the DUT in its linear region. To improve the EVM performance, we reduce the PAPR and the ACPR of the transmitter, so that the developed system consists of the stages of clipping and filtering, peak cancellation, and peak windowing. In this work, the CFR-clipping technique has been exploited to reduce the PAPR values OFDM transmission by minimizing the EVM with a constraint on PAPR for an RF PA operating at 2.40 GHz. Additionally, this involves a very sharp CCDF curve during the PAPR evaluation. The clipping principle is based on detecting peak voltage based on a defined reference related to the linear region of a PA with a signal x(n) for a power defined by $P_{ref}$.

(1) $$cp\left(n\right)=\left\{\begin{array}{cc} x\left(n\right)\frac{P_{ref}}{\left|x(n)\right|} & \;\mathrm{if}\;\left|x(n)\right|\geq P_{ref}\\ 0 & \;\mathrm{otherwise} \end{array}\right.$$

The CFR and clipping procedures introduce a filter to compensate the out-of-band component related to cp(n), the signal can be defined as follows:

(2) $$x_{c}\left(n\right)=x\left(n\right)-filter\left[cp\left(n\right)\right],$$

CFR and clipping together with a method of linearization can significantly improve the DPD performance required during a hardware implementation. The PAPR can be reduced based on CFR and trimming process under the same structure. The linearization of a transceiver system involving CFR and clipping method can be expressed as:

(3) $$\begin{array}{c} cp\left(n\right)=\gamma_{\alpha }\left[x\left(n\right)\right], \end{array}$$

(4) $$\begin{array}{c} u\left(n\right)=\gamma_{\beta }\left[x\left(n\right)\right], \end{array}$$

where x(n) can be defined as the input signal, cp(n) is the CFR function and u(n) depicts the linearized signal. The clipped signal $u_{c}\left(n\right)$ is expressed as:

(5) $$u_{c}\left(n\right)=u\left(n\right)-up\left(n\right)=\gamma_{\theta }\left[u\left(n\right)\right],$$

where the target to be achieved is defined by θ=β−α. It should be noted that θ is represented by a set of parameters of β and α. A variation of the clip and filter approach is the peak cancellation, $\gamma_{\alpha }$ and $\gamma_{\beta }$ are a set of coefficients that relate the input signal to the ILA output and the clipped signal; z(n) is defined by the attenuation associated to the value of the maximum input peak power between the maximum value of the system power multiplied by the amplified signal, z(n) is used in the coefficient estimation process for the ILA approach. The CFR and linear correction signals are separated, the peaks in the linear signal, measured at the channel filter output, are detected. A band-limited pulse is generated at each peak location. The magnitude of the pulse is set equal to the peak in excess above the clipping threshold, the phase of the pulse is the same as the phase of the linear signal at the peak, a filter determines the response of the pulse, the pulse sequence filtered is subtracted from the linear signal to produce a band-limited signal with the desired PAPR calculates as,

(6) $$\mathrm{PAPR}\left(\mathrm{dB}\right)=10\log_{10}\frac{P{\left(t\right)}_{peak}}{P{\left(t\right)}_{rms}}.$$

### 2.2. CFR Algorithm to Single-Band Signals with PAPR Target

**Figure 3:**
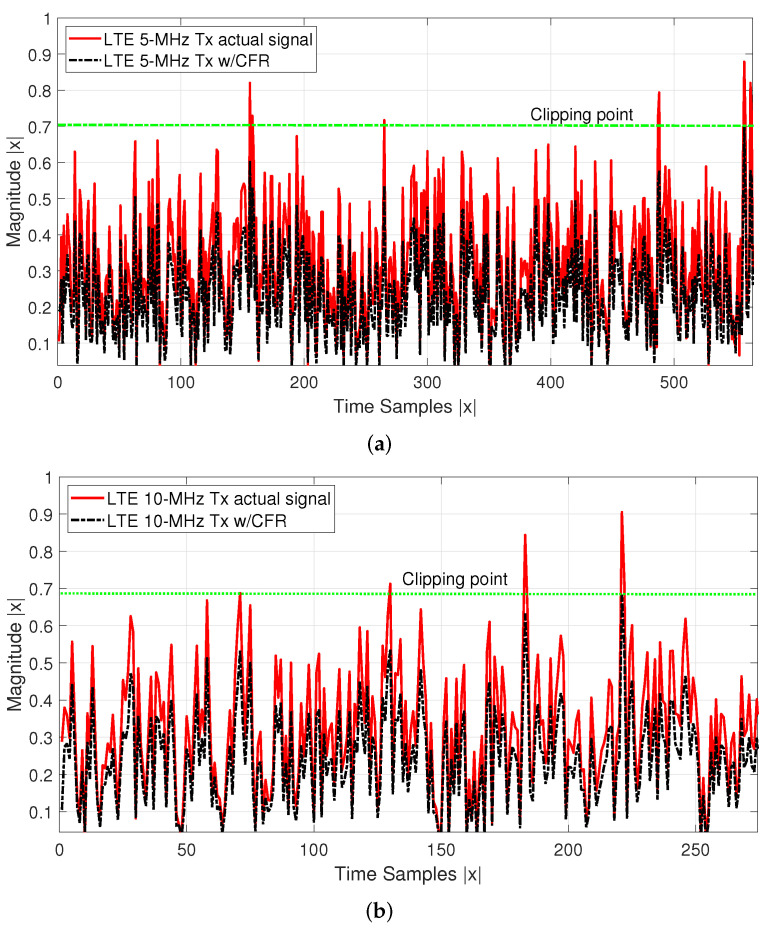
(**a**) LTE-5 and (**b**) 10-MHz actual signal vs. CFR signal for time samples.

**Figure 4:**
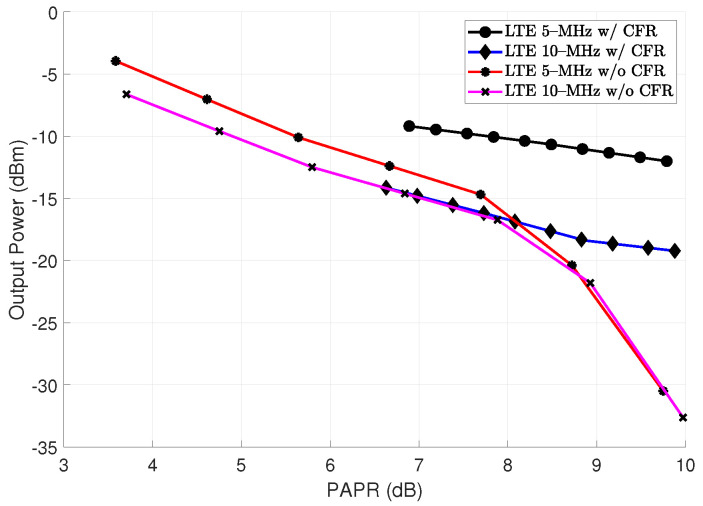
LTE 5–MHZ and 10-MHz signal PAPR vs. output power level with and without CFR applied.

The peak windowing for CFR lowers the PAPR, attenuating the waveform for a short time interval surrounding a peak. Once a peak is detected in the waveform, the peak-centered window function attenuates the waveform at the peak and its neighboring samples. The window function will typically have a Gaussian shape that is time domain truncated. Other suitable functions include the Hann, Hamming, and Kaiser windows. The peak with waveform window $x_{w}$ is:

(7) $$x_{w}=\left(1-c^{\prime }\left(n\right)\right)x\left(n\right),$$

where x(n) is the input signal, and $c^{\prime }\left(n\right)$ is the clipping algorithm defined by the following equation:

(8) $$c^{\prime }\left(n\right)=\sum_{m}\gamma_{m}\sum_{\tau }g\left(\tau \right),\delta_{peaks}\left(n-\lambda_{m}-\tau \right)$$

where $\lambda_{m}$ is the location of peak *m*, $\delta_{peaks}$ is the location in time of the detected peak, g(τ) is a Gaussian window and $\gamma_{m}$ can be expressed as:

(9) $$\gamma_{m}=\max\left(1-\frac{L}{\left|x\right|},0\right).$$

where *L* is the desired level of clipping. Algorithm 1 shows the pseudocode of the CFR-based technique to evaluate the PAPR performance. It should be noted that the purpose of developing a CFR system is to reduce PAPR levels of at least 3 dB in order to compare against DPD methods and alternative spectral improvements works lately reported. The CFR algorithm implementation is used for a 2.40 GHz single carrier frequency for 64–QAM digital modulation multiplexed by OFDM. In Algorithm 1, it can be observed that the inputs are described as x(n) in which a signal cp(n) is obtained based on a reference power. This signal is filtered to compensate for the out of band components and is obtains an $x_{c}\left(n\right)$ and obtain a clipped signal cp(n), so that based on Equation (5) a comparable signal is obtained with hardware-based linearization methods.
**Algorithm 1:** CFR algorithm with PAPR target.**Input**: n≥0**Output**: $y=x^{n}$y←1;X←x;
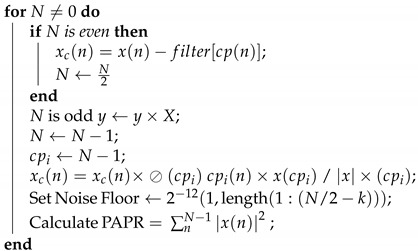


Table 1 shows the relationship obtained with each CFR test performed. It can be seen that the EVM obtained based on the clipping process varies in each stage, given the deterioration of the vector to be analyzed. The variation due to the improvement of the EVM can be related to corrections of the I/Q umbalance, phase gain, DC offset, among others. Figure 3 shows the actual LTE 10-MHz signal when comparing it with the resulted clipping signal both estimated using a frame with N= 76,800 complex-valued data samples while peak integrity is guaranteed. In Figure 4, the PAPR achieved in the transmitter Tx due to the clipping and filtering stages is shown on the x-axis. An example of an algorithm with lower computational complexity analysis related to CFR and DPD modeling with spline-interpolation coefficients LUT, which aims to preserve a learning rule based on parameters is reported in [24]. Equation (6) shows the $P{\left(t\right)}_{peak}$ represents the peak measured at the fundamental for the LTE implementations in this case of −9.18 dBm for LTE 5-MHz and −14.04 dBm for LTE 10-MHz, in addition to the values $P{\left(t\right)}_{rms}$, for each transmitted symbol of 64–QAM digital modulation under OFDM multiplexing.

## 3. Implementation and Measurement

### Experimental Setup Testbed

**Figure 5:**
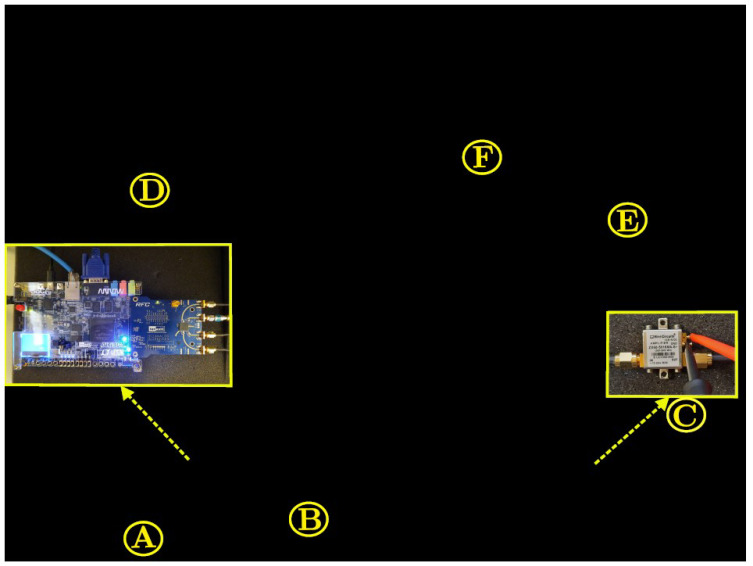
Experimental testbed photograph. Relevant instruments depicted as follows. (**A**): Altera Cyclone V FPGA SoC-Kit. (**B**): AD9361 RF Agile Transceiver working at 2.40 GHz center frequency. (**C**): High isolation amplifier Mini-Circuits ZX60-5916MA+. (**D**): Power Supply GW INSTEK GPS-3303. (**E**): Display HOST PC-MATLAB. (**F**): Spectrum Analyzer SIGLENT SSA3032X.

The experimental setup considers the acquisition of real-valued data from several run tests to calculate CFR, PAPR, and ACPR signal measurements considering the DUT. Figure 5 shows the developed SDR testbed used to obtain the experimental results, it consists of a radio frequency system-on-chip with a 2×2 board dual channels of the I and Q reception quadrature signals, these pass through a type finite impulse response (FIR) filtering stage to be handled by an adjustable internal sampling rate to produce a 12–bit output signal up to 122.88 MS/s according to the signal bandwidth requirements. The DUT considers a ZX60-5619MA+ amplifier with 50 ohms output impedance, which is used with 5 V bias with a typical gain of 17.27 dB for 2 GHz band applications. A libiio library is run on this embedded Linux to provide a full-duplex communication between the data from MATLAB and the FPGA hardware modules. For the RF transceiver board AD9361; MATLAB sends the data to the FPGA; the data link is made over Ethernet between the ADI Hardware system connected to the FPGA-SoC platform that runs on ADI Linux. The RF transceiver performance provides a narrow range from 2.4–2.5 GHz to support signals up to 56-MHz using a dual 12–bit DACs with a synchronization clock set at 245.76 MHz. The up-conversion path uses an I/Q modulator driven by an internal local oscillator (LO) operating at the frequency of 2.40 GHz to generate the RF signal. The LO is provided internal and operates from 47 MHz to 6.0 GHz.

To perform the test and evaluation of the proposed methods, separate experiments are carried out. The first measurement tests are conducted with the identification of CFR, ACPR, and PAPR, to estimate the values in a feedback iterative procedure. The measurement system includes the FPGA, RF transceiver dual-channel sub-6 GHz, wideband amplifier ZX60-5916MA-S+ that feature a typical gain ≈ 17.33 dB biasing with 5 V. At the feedback path, a receiver is used to digitize the down-converted signal using an I/Q demodulator operated at a sampling rate of 245.76 MHz, while an interpolation filter is applied as data rate is set for a given bandwidth. Here OFDM symbols and subcarrier EVM (%) against subcarriers in the frequency domain and symbols in the time domain are described. In this case, the EVMs are calculated for each carrier within a frame and the peak values are displayed in the 3D graphic, the EVMs for each carrier are shown for 64–QAM using frequency division duplex (FDD). In this implementation, the EVM performance is evaluated based on a reference measurement channel (RMC) for a downlink test. The digitally modeled signal to measure the peak and root mean square (RMS) EVM average of the input signal frames.

For the output DUT amplifier measurement, a SIGLENT SSA3032X was carried out for analysis in the frequency domain. In this case, the setup for the analysis of EVM, PAPR, and ACPR are developed in MATLAB through the LTE toolbox and the RF Blockset models for Analog Devices RF Transceivers. The data input is sent from the host computer, and the ARRadio card setting tests operates with an AD9361 RF transceiver using a carrier frequency 2.40 GHz and an LTE 10-MHz with a sampling rate of 245.76 MHz and 122.88 for the LTE 5-MHz and performed for a IFFT size of 1048.

**Figure 6:**
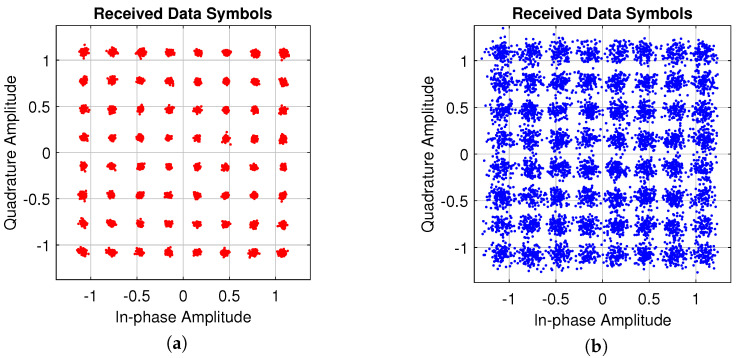
Constellations diagram for the actual transmitter LTE 10-MHz 64–QAM signal and CFR scale factor, (**a**) actual 64–QAM 10-MHz signal, and (**b**) receiver signal.

**Figure 7:**
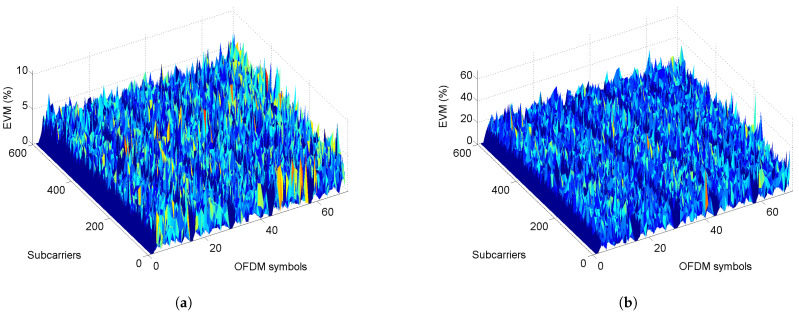
EVM (%) for the input and output signal, before and after applying a scale factor: (**a**) actual 64–QAM 10-MHz signal, and (**b**) receiver signal.

Therefore, for our particular results in the transmission path, the EVM is preserved from a linear target dataset, using the complex baseband input signal x(n) that considers an interference-free situation and calculated at the receiver signal to obtain a representation of the impact of hardware impairments. As shown in the Figure 6 and Figure 7, we evaluate two target performance tests: (*i*) the best EVM used $I_{bst}\left(n\right)$ to preserve metrics, which is saved as an actual transmitter signal, then we compare (*ii*) after clipping conditioning. If we consider the first test with negligible hardware impairments, thus, the EVM should only be determined by the receiver noise. In Figure 7, the system performance evaluated is shown with its EVM associated at transmitter and receiver, and the EVM is sampled and compared against the OFDM symbols. As it is well known, the calculated EVM is sensitive to any noise and signal distortions that are reflected in its amplitude and phase components, thus a further correction can be applied using DPD. As a rule, when calculating the EVM, systematic errors (i.e., the shift of the constant component of current or voltage, phase, I/Q or amplitude imbalance, etc.) are not taken into account and require preliminary correction. However, such correction is not full. On the other hand, a very important role has the distance between sources of signals in angle coordinates. The EVM is calculated from its percentage as the square root of the power ratio between mean error vector to the mean reference vector power for a given number of samples $N=2^{12}$ as follows:

(10) $$\begin{array}{c} \mathrm{EVM}\left(\%\right)=\sqrt{\frac{\frac{1}{N}\sum_{n=1}^{N}|I_{bst}\left(n\right)-I_{meas}\left(n\right){|}^{2}+\sum_{n=1}^{N}|Q_{bst}\left(n\right)-Q_{meas}\left(n\right){|}^{2}}{\sum_{n=1}^{N}|I_{bst}\left(n\right)+Q_{bst}\left(n\right){|}^{2}}}\times 100 \end{array}$$

It consists of two transmitter cards that enable the upconversion and filtering through the AD9361 transceiver before passing it to the ADC in the receiver processing. To estimate PAPR and the signaling condition, it is crucial to evaluate the TX-RX overall performance, in [25] it is defined as a weighted cubic-spline approach as a proper alternative and multivariable analytical modeling to reinforce the spectral efficiency. The purpose of using this development board is to integrate RF applications with systems capable of being programmed with variable bandwidths for LTE applications. To evaluate the PAPR obtained, the CCDF is used to represent the probability that the envelope signal exceeds a defined threshold in the implementation stage. In this evaluation, a PAPR target reduction of at least 3 dB is defined, the CFR process-clipping based is introduced in the implementation stage for LTE signals evaluation, under OFDM multiplexing, in this case the PAPR is calculated with the real-valued samples data as:

(11) $$\mathrm{PAPR}\left(n\right)=\frac{{\left|x(n)\right|}^{2}}{\frac{1}{N}\sum_{n}^{N-1}{\left|x(n)\right|}^{2}}$$

CCDF is related to the PAPR calculation based on the relation:

(12) $$\mathrm{CCDF}\left(\%\right)=P_{t}\left(\mathrm{PAPR}>P_{ref}\right),$$

where $P_{t}$ provides information on the amount of time the signal spends at or above a specific power level, and $P_{ref}$ is denoted as the PAPR target. The setup system is calibrated so that a synchronized signal is applied properly to the amplifier input. Note that we set a calibration power for power peak and average levels at the amplifier output after each iteration in order to obtain several CFR factors. Figure 5B shows the transceiver that operates in the band; the ARRadio card integrates an AD9361 RF transceiver to operate in an range of 2.4–2.5 GHz. Once the sample rate and RF bandwidth have been established, the signal to be implemented in MATLAB is designed as shown in Figure 5E, performing with the 64–QAM modulation. It can observe the processes of collecting LTE parameters according to a LTE mode selected, generate LTE signals using the LTE system toolbox, the transmission and reception using (AD-FMCOMMS3-EBZ) RF transceiver board and the MATLAB library to pre- and post-processing of captured real-valued data.

## 4. Experimental Results

### 4.1. CFR Measurement Results with LTE Signals

**Figure 8:**
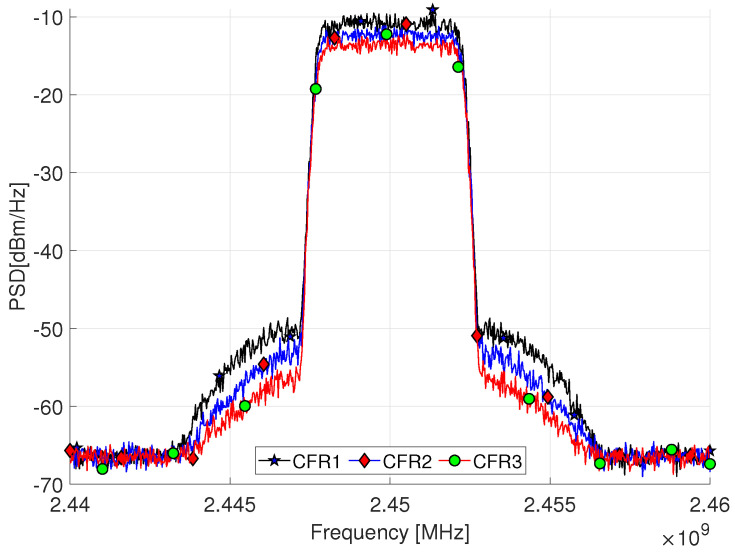
PSD of LTE 5-MHz OFDM waveform signal with residual spectral regrowth after CFR using three scale factors.

**Figure 9:**
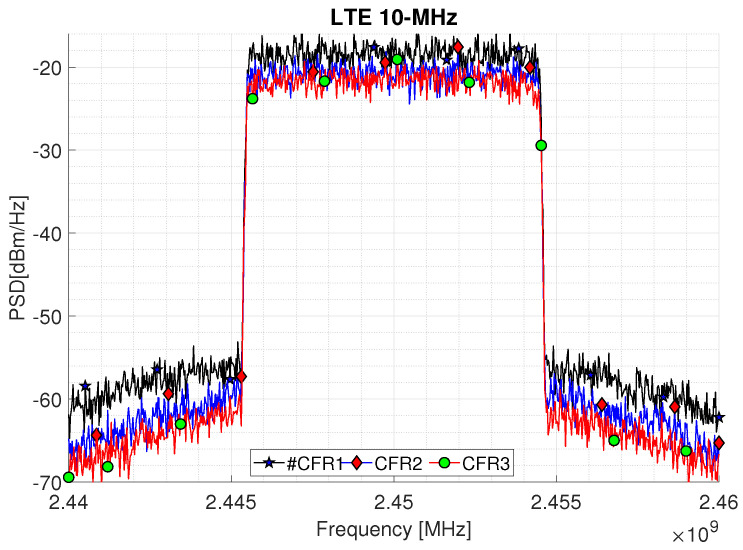
PSD of LTE 10-MHz OFDM waveform signal with residual spectral regrowth after CFR using three scale factors.

Comparing Table 2, it can be seen that CFR reduce the PAPR of the developed system. The PA measurements as DUT include the analysis of the PAPR and ACPR based on a variation of the clipping in the CFR stage. Figure 8 and Figure 9 depict the power spectral density (PSD) performance for the CFR factors as summarized in Table 2 for the 5 and 10-MHz bandwidth signals, respectively. Figure 8 shows the ACPR for three CFR factors, where a PAPR at the Tx shows an improvement of 2.94 dB for a LTE 5-MHz. While an improvement of 3.30 dB is obtained for the LTE 10-MHz as shown in Figure 9, which represents a trade-off between the PAPR and ACPR controlled by the CFR threshold and *n* clip factor; this is represented by Equations (1)–(9). The actual signal exhibits a PAPR of around 10.87 dB with an output power −39.43 dBm for an LTE 5-MHz. In this sense, it is considered that the CFR can be targeted from PAPR level outputs from 6.88 to 9.82 for a LTE 5-MHz and from 9.87 to 6.57 for a LTE 10-MHz. This conditions represents a variation that derives directly from the clipping threshold defined by Equation (9), where *L* is the desired level of clipping, additionally a CFR of Algorithm 1 is shown with a PAPR target established as a means of optimizing the process.

#### LTE Proposal Study Case

**Figure 10:**
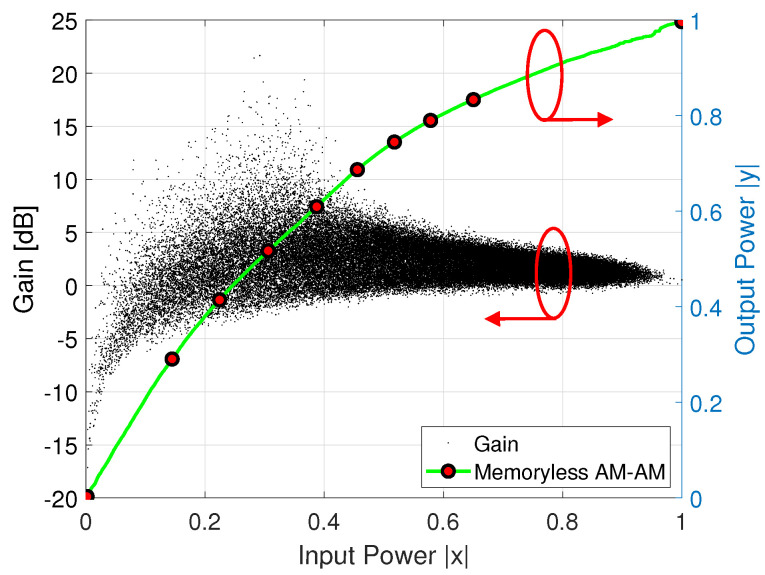
Gain for the normalized input and output signal vs. memoryless AM–AM characteristic.

**Figure 11:**
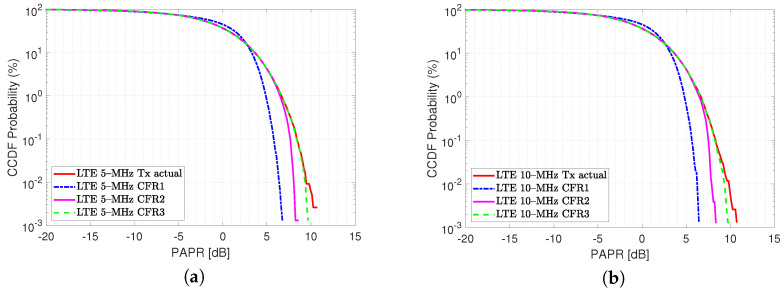
CCDF of OFDM signal; (**a**) CFR iterative factor for LTE 5-MHz waveform and (**b**) LTE 10-MHz waveform.

In this work, the performance of LTE signals of 5 and 10-MHz bandwidths are evaluated for commercial LTE applications. For the evaluation a single band, a LTE signal is used as the input stimulus to drive a Mini-Circuits ZX60-5916MA+ amplifier. In this case, the signal is measured in the Tx under a 64–QAM modulation scheme for both bandwidth cases. In order to highlight the three CFR factor cases related with its residual spectral regrowth, its ACPR associated is calculated at lower/upper ±2.5-MHz frequency offsets for the LTE 5-MHz and ±5-MHz for the LTE 10-MHz. Figure 10 shows the gain distortion for a given input power using an LTE 10-MHz stimulus in the DUT for a normalized output power using the entire 76,800 samples. To obtain a memoryless model for given input power, it is swept by iteratively increasing the scale factor to obtain the best AM–AM characteristic, which aims to preserve the useful data points in the saturation level. The clipping factor is then applied for three cases in the LTE actual signal and it is shown in Figure 7, where a reduction can be targeted, noting that after applying the CFR, the PAPR value is reduced from around 4 dB of reduction as depicted in the probability function shown in Figure 11.

(13) $$\begin{array}{cc} \mathrm{ACPR}\left(\mathrm{dB}\right)≜ & 10\log_{10}\left(\frac{\sum_{n=1}^{N}\cdot |{y|}^{{}_{2}}\cdot e^{\left(j\omega n\right)}}{\sum_{n=1}^{N}\cdot {\left|y_{\mathrm{low}}\right|}^{2}\cdot Ye^{\left(j\omega_{1}n\right)}+\sum_{n=1}^{N}\cdot {\left|y_{\mathrm{upp}}\right|}^{2}\cdot Ye^{\left(j\omega_{2}n\right)}}\right) \end{array}$$

Figure 11 shows the CCDF of the input signal after three clipped factors represented by the transmitted signal, respectively. As can be seen, the CFR stage reduces the PAPR of the input signal from 11 dB to 5 dB. This process impacts on an improvement in the EVM, the PAPR distribution of the LTE PA input signal with a desired PAPR of 3 dB, it can be observed that after 10 CCDF iterations reach the probability between $10^{-3}$ and $10^{-4}$, in this condition, the optimal PAPR level is reached.

### 4.2. Determining the Digital Predistorter Model

For the predistorter, a behavioral model is used in the first instance. In this case, the ILA technique is used to correct the residual spectral regrowth, this correction is validated for a mathematical model, and the power reduction in the sidebands is evaluated, in addition to the EVM performance. For the residual ACPR spectral regrowth after CFR, a modeling and linearization stage based on the polynomial model with memory for the purpose of the AM–AM curve of the device is developed. The derived modeling procedure generates the amplitude relationship for a modulation scheme under test, in this work are performed for quadrature phase-shift keying (QPSK), and LTE digital multiplexing under 64–QAM modulation.

(14) $$y\left(n\right)=\sum_{k=1}^{K}\sum_{m=0}^{M-1}=d_{k,m}y_{d}\left(n-m\right){\left|y_{d}\left(n-m\right)\right|}^{k}.$$

Based on the peak gain of the system, the $y_{d}\left(n\right)$ is determined, which is expressed by the following Equation (15):

(15) $$y_{d}\left(n\right)=y\left(n\right)/G\left(n\right),$$

where G(n) is based on the maximum values of x(y) and y(n), as expressed in Equation (16).

**Figure 12:**
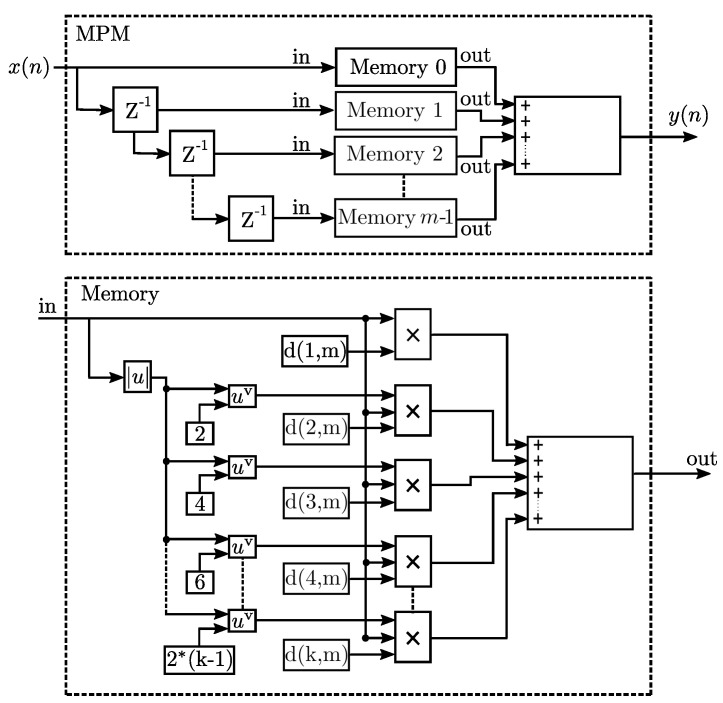
Block diagram for the polynomial model with memory.

(16) G(n)=y(n)max|x(n)|/max|y(n)|,

As mentioned before in a first approach the CFR uses the baseband LTE signal to reduce the clipping in the signal, thus improving the PAPR. However, the residual spectral regrowth can be also mitigated by applying DPD. The Volterra series variation provides an approximation on nonlinear effects of the short-term memory effects of a DUT under test and the linear time-invariant (LTI) stage. In this sense, a structure derived from the Volterra Series is chosen as the memory polynomial model (MPM) to introduce in a single-stage the static and non-linear functions to represent the AM–AM recorder [26,27]. In this case, by taking into account only the diagonal kernels, the MPM makes an adequate approximation of the model. The structure used by the MPM is representative of the Equation (17):

(17) $$y\left(n\right)=\sum_{k=1}^{K}\sum_{m=0}^{M}=a_{m,k}x\left(n-m\right){\left|x\left(n-m\right)\right|}^{k-1},$$

where, $a_{m,k}$ represents the coefficients related to the static and dynamic part of the model, x(n) the sampled and discretized input signal, y(n) the output signal, *M* the short-term memory level of the system, and *K* the order of the polynomial to represent the static part. The coefficients to determine the behavior DUT are determined by the least-squares method (LSM). The MPM stages divided into two sections are represented by the Figure 12. DPD uses digital processing and is established in the FPGA domain, the high-frequency signal that operates in the 2 GHz band for the down-conversion of the development card and is established as input y(n) of the DPD system. Subsequently, a CFR stage will be implemented based on a three-level cut stage for PAPR reduction. The system is implemented for a ZX60-5916MA-S+ with a typical gain ≈ 17.33 bias with 5 V for commercial LTE applications and QPSK testing.

**Figure 13:**
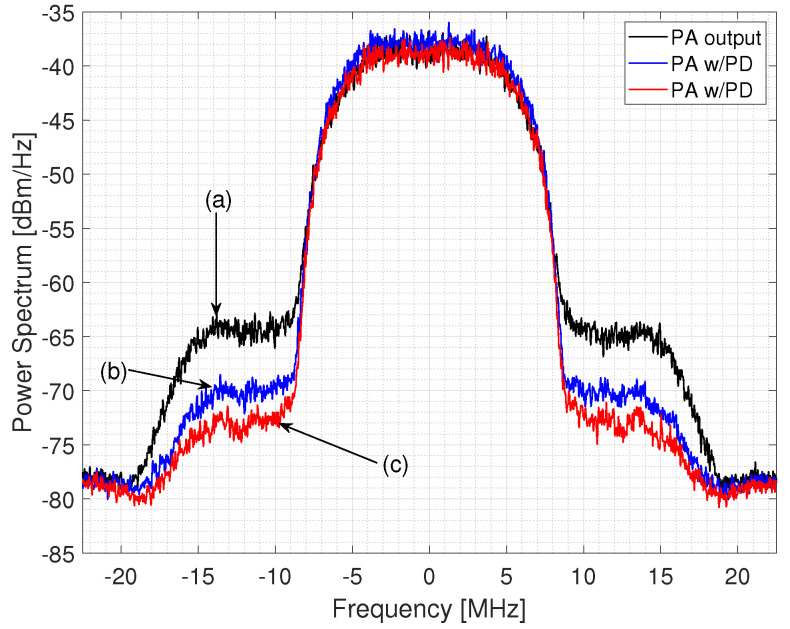
Spectral regrowth of the QPSK 18-MHz; (**a**) PA Output (Saleh model), (**b**) with PD (K=4,M=1), (**c**) with PD (K=6,M=2).

Considering the closed-loop behavioral modeling process that was shown in Figure 2, it provides the general diagram of the indirect learning architecture (ILA) for the amplifier as DUT. In this procedure, we estimated the x(n) signal introduced before/after the CFR stage. In this case, based on Equation (2), an $x_{c}\left(n\right)$ is obtained, this is the block where the peak cancellation and the peak window are performed, the resulting signal $x_{c}\left(n\right)$ is introduced in an iterative procedure to the ILA linearization block to obtain a u(n) that will be introduced to the DUT. Therefore, the actual input signal x(n) can be swept to an input stimulus to obtain the output signal y(n). In the learning stage, the coefficients of the linearization stage are calculated based on a least-squares estimation linear regression, and a comparison is made with the AM–AM characteristic of the device. We implement a Saleh model to set a nonlinearity parameter, with an input scaling (dB) whose parameter scales the input signal before the nonlinearity is applied [28]. The AM–AM parameters, α, and β are used to compute the amplitude gain for an input signal as follows

(18) $$F_{\mathrm{AM}-\mathrm{AM}}\left(u\right)=\alpha_{1}\left(u\right)/\left(1+\beta_{1}u^{2}\right),$$

where *u* is the magnitude of the scaled signal, the AM–AM parameters, α, and β, are used to compute the phase change for an input signal using the function depicted by Equation (19).

(19) $$F_{\mathrm{AM}-\mathrm{PM}}\left(u\right)=\alpha_{2}\left(u\right)/\left(1+\beta_{2}u^{2}\right).$$

where *u* is the magnitude of the input signal. Note that the AM–AM and amplitude to phase conversion (AM–PM) parameters, although similarly named alpha and beta, are distinct. For the linearization of the MATLAB virtual amplifier (Saleh model), ILA is used in Figure 13; the spectral growth of the received signal is observed as a result of the use of the amplifier where DPD is not used as before the PA, while in Figure 13 we observe the spectrum of the received signal where its power values decrease in the bands thanks to using the DPD with ILA with m=2 and k=6, that is, three memory stages with a depth of 6, that is, with 18 coefficients the polynomial model. The behavior modeling for derive the predistorter stage is carried out starting from the device characteristics, measurement, and modeling of an RF PA, which considers memory effects and nonlinearity; where the function of the input and the output are interchanged as shown in Equation (14), to model the nonlinear function of the linearization as is represented in Equation (15). For the modeling design, the sweep of the input tone is taken into account, the measurements of the modeling of the AM–AM conversion curve is the basis for the calculation of the coefficients $d_{k,m}$, which generates a new input $y_{d}\left(n\right)$ based on the DUT peak gain y(n) is represented by the Equation (14) where the even and odd coefficients based on the LSE method are taken into account. The predistorter effectiveness for two configurations of parameters *k* and *m* are summarized in Table 3.

### 4.3. Discussion

The present work represents a platform development and methodology based on the ILA of commercial RF PAs with LTE applications. It is based on sweeping the signal from the AD9361 dual transceiver using a virtual PA for QPSK and 64–QAM modulation. The achieved metric to evaluate the system is 8 dB of spectral reduction achieved. The developed system reaches a value of 7.5% of EVM, which is the primary specification that quantifies the performance of digital modulation. The system represents a preliminary platform for DPD applications based on LTE digital multiplexing schemes operating in the 2.5 GHz band. Its operating range allows modeling and linearization in licensed and unlicensed bands and bandwidths of 200 KHz to 56 MHz. Table 4 shows a comparison of the application ranges in frequency; the developed system explores an integration of CFR-clipping that can be joint helps to increase the PA efficiency, while simultaneously provide a better linearity performance, for wide-range applications from 47 MHz–6 GHz. The CFR-clipping method before the DPD stage allows achieving PAPR reductions compared with seminal works. Most of the works summarized in Table 4 are based on a signal baseband generation defined as CW Source, VSG or simulation, these approaches involve a limited transceiver system, and the tuning is limited for specified applications. Table 5 shows the wide range of digital applications to achieve spectral reduction and improvement with multi-carrier systems. OFDM and W-CDMA applications predominate towards 5G, in addition to bandwidths of 5–20 MHz. It can be seen that the developed work is an integration of a system based on CFR technique-clipping based in closed-loop iterative process, which achieves a reduction of up to 3.3 dB in PAPR. The system is based on more than 60 OFDM symbols for each of the 600 subcarriers, in addition to an EVM study (%) with an improvement from 5.7% to 31.39% with the CFR approach of 5-MHz, in addition to an EVM improvement (%) from 5.46% to 39.21% with CFR 10-MHz.

In this work, a platform was developed to validate the robustness of the CFR-clipping algorithm with experimental results, they can be scaled in various digital multiplexing schemes such as OFDM or W-CDMA with multi-carriers, where the systematic tuning can be automated controlled for the whole SDR system.

The developed algorithm is dependent on the digital domain, unlike the other reported works [26,28]; these approaches are based mainly on vector signal generator (VSG), or vector signal transceiver to generate the I/Q baseband data samples [18,24], which is more expensive test equipment, and the signals are predefined. In this context, our proposed CFR-clipping system can be fully controlled for LTE 5-MHz and LTE 10-MHz applications, from 47-MHz to 6-GHz. This algorithm links a CFR-clipping stage before a DPD block. The system can control the power spectra of carrier frequency by varying the power scale factor in the board, which is associated with CFR and clipping procedures that are detailed in the subsection CFR Model Identification with Joint PAPR Clipping. This work evaluates different CFR-clipping levels to improve the precision and spectral reduction of a DPD technique integrated with the CFR-clipping algorithm.

In this developed work, the CFR algorithm it is validated with a commercial RF PA from Mini-Circuits ZX60-5916MA+ for LTE applications as a DUT in the platform. The on-board transmit power monitor can be used as a power detector, enabling highly accurate TX power measurements; in the case of the compared works, it is not so precise if a VSG is used as the input device instead of a development board. This is the primary key and purpose of utilizing a software defined radio (SDR) board for scalability between applications for sub-6GHz frequency bands.

## 5. Conclusions and Future Work

In this work, a clipping-based algorithm was developed to reduce the PAPR of OFDM signals using a FPGA-based system. The proposed CFR algorithm is experimentally validated in terms of PAPR, ACPR, and residual spectral regrowth performance improvement. The conditioning waveform with the simplest approach clip and filter method not only aids to take clipped points, but also from the actual signal preserve its feature when a factor clipping is applied. The CFR model identification with joint PAPR clipping is applied to operate the PA with better energy efficiency in order to affect the amplified signal to introduce in its linear region. A reduction of 3.3 dB is obtained for the transmitter in the 10-MHz and 2.93 dB for the Tx for a 5-MHz bandwidth. The CFR algorithm performance is done on a commercial amplifier for 64–QAM OFDM LTE applications with bandwidths of 5-MHz and 10-MHz with an average increase of the EVM obtained as 39.94% and 12.82% for 10-MHz. For future work, we expect to implement in the FPGA system robust algorithms based on the direct and indirect learning architectures, to identify the coefficients for the real-time signals and explore new DPD systems, with its CFR algorithm associated under a parameters preservation approach.

## Figures and Tables

**Table 1:** EVM performance for LTE 5-MHz and LTE 10-MHz.

Meas	LTE 5-MHz wo/ CFR	LTE 5-MHz w/CFR	LTE 10-MHz wo/ CFR	LTE 10-MHz w/CFR
Min EVM (%) Peak	3.50%	19.75%	4.13%	22.48%
Max EVM (%) Peak	9.20%	49.14%	9.59%	61.69%
Peak-to-average EVM (%)	4.56%	30.62%	5.67%	31.03%
Minimum EVM (%) RMS	1.59%	10.95%	2.16%	11.50%
Maximum EVM (%) RMS	4.18%	16.89%	4.51%	17.64%
Average of RMS (%)	1.89%	13.32 %	2.60%	14.12%

**Table 2:** PSD signal results after applying three CFR factors for the LTE-5 and LTE 10-MHz.

LTE 5-MHz	PAPR Tx w/CFR (dB)	ACPR Lower & Upper (dBc)	$P_{\mathrm{out}}$ (dBm)
CFR1	6.88	−60.50/−60.56	−9.18
CFR2	8.52	−62.08/−62.09	−10.39
CFR3	9.82	−63.51/−63.59	−12.04
**LTE 10-MHz**	( **PAPR Tx w/CFR** ( **(dB)**	( **ACPR Lower & Upper** ( **(dBc)**	**P_out_** **(dBm)**
CFR1	6.57	−58.15/−60.56	−14.04
CFR2	8.87	−62.53/−62.49	−18.39
CFR3	9.87	−64.57/−63.59	−19.22

**Table 3:** Predistorter effectiveness for the QPSK 18-MHz signal through a virtual PA (Saleh model).

Case	RMS EVM (%)	Avg EVM (dB)
PA output	8.4	−21.5
With PD k=4 m=1	8.1	−21.9
With PD k=6 m=2	7.6	−22.4

**Table 4:** Comparative table for the CFR technique, linearization method and signal generation.

Related	Fundamental	Predistortion	Baseband Signal
Work	Frequency	Technique	Generation
This work	47 MHz–6 GHz	CFR-Clipping and	FPGA RF Agile Transceiver
		Memory polynomial model	
Xiaofan Chen, et al. [7]	1.9–2.6 GHz	2-D power distribution reshaping	CW Source
Mehdi V. Amiri, et al. [14]	Not specified	new spectrum constrained-CFR	VSG
R. Neil Braithwaite [15]	1952–1965 MHz	DPD-CFR	VSG
S. Wang [16]	3.3–3.8 GHz	clipping-and-bank	Arbitrary waveform
		filtering	generator
S. Mohammady, et al. [17]	Not specified	DPD-least squares (LS)	FPGA-Transceiver
		optimization	
K. Anoh, et al. [19]	Not specified	Clipping signal	Not specified
S. Gökceli, et al. [20]	9 kHz–3.5 GHz	Iterative clipping	VSG
		and weighted error filtering	
L. Yang and Y. Siu [21]	Not specified	Clipping and filtering	Monte Carlo Simulation
H. Abdelali, et al. [22]	Not specified	Tone Reservation	Simulated WiMax 802.16a
		clipping method	system
J. Chatrath, et al. [26]	2.2 GHz	MPM Modeling	VSG
R. Sanjika and D. Kurup [27]	3.44–3.60 GHz	Not specified	Not specified
S. Mohammady, et al. [29]	Not specified	Clipping-Filtering and	Not Specified
		Peak-Shrinking Interpolating	

**Table 5:** Comparison of spectral improvements and applied techniques for digital applications.

Digital Application	Achieved Precision and Spectral Improvement	Spectral Reduction Technique
LTE 1.4 MHz [26]	−36.9 dB NMSE	Augmented MP
QPSK, 8-PSK and 16-QAM 10/20 MHz [27]	−35.62 10 MHz, −37.40 20 MHz	ANN, Rapp model
LTE, W-CDMA [29]	PAPR 4 dB	CFR techniques-Clipping based
16 QAM and 64 QAM, OFDM [17]	PAPR 4 dB	CFR techniques-Clipping based
OFDM, WiMax 802.16e [20]	PAPR not specified	Clipping based
OFDM, WiMax 802.16e [22]	PAPR not specified	Clipping based
OFDM [21]	PAPR not specified	iterative clipping noise
OFDM [19]	PAPR 9.5 dB	Iterative clipping
	and BER improvement	and filtering
Not specified [16], general purpose	PAPR 3 dB	CFR and DPD neural networks (NN) based
W-CDMA multi-carrier [15]	PAPR 8.6 dB	CFR and envelope clipping
OFDM, 64–QAM [14]	PAPR 8.6 dB	CFR and envelope clipping
LTE 10-MHz [7]	0.3 to 0.8 dB Power improvement	1D-CFR
OFDM, LTE-10 and 5-MHz [This work]	PAPR 3 dB (5-MHz)	CFR technique
	PAPR 3.3 dB (10-MHz)	Clipping based

## Data Availability

Not applicable.
